# Supplementary material for: MetaRibo-Seq measures translation in microbiomes
Source: Nat Commun. 2020 Jun 29;11:3268. doi: 10.1038/s41467-020-17081-z (PMC7324362; doi:10.1038/s41467-020-17081-z)
Supplement: Supplementary file 10 — Supplementary Data 7 [file 41467_2020_17081_MOESM10_ESM.zip › File2/Confidence_VeryHigh_Taxonomy/186971_out.krona.html]

Javascript must be enabled to view this page.

members
magnitude
magnitudeUnassigned
count
unassigned
taxon
rank

186971\_out

7

superkingdom
2
7

7
1239
phylum

6
186801
class

186802
6
order

1
31979
family

genus
1485
1


SRS075821\_contig\_number\_contig-100\_27974.27975
species
1896983
1

family
1
186803


SRS042131\_contig\_number\_contig-100\_1060.157085
species
712976
1

family
4
541000


SRS063518\_contig\_number\_14875
216851
1
genus
4

3
853

SRS042628\_contig\_number\_contig-100\_654.655SRS143181\_contig\_number\_contig-100\_2158.85718SRS147377\_contig\_number\_9435
species


SRS1041140\_contig\_number\_5611
species
1
1263007
